# A Review of Transcriptomics and Metabolomics in Plant Quality and Environmental Response: From Bibliometric Analysis to Science Mapping and Future Trends

**DOI:** 10.3390/metabo14050272

**Published:** 2024-05-08

**Authors:** Qi Yan, Guoshuai Zhang, Xinke Zhang, Linfang Huang

**Affiliations:** Key Lab of Chinese Medicine Resources Conservation, State Administration of Traditional Chinese Medicine of China, Institute of Medicinal Plant Development, Chinese Academy of Medical Sciences, Peking Union Medical College, No. 151, Malianwa North Road, HaiDian District, Beijing 100193, China; yanqi@implad.ac.cn (Q.Y.); zhangguoshuai@implad.ac.cn (G.Z.); zhangxinke@implad.ac.cn (X.Z.)

**Keywords:** transcriptome, metabolome, bibliometric analysis, research stream, plant quality, environmental response

## Abstract

Transcriptomics and metabolomics offer distinct advantages in investigating the differentially expressed genes and cellular entities that have the greatest influence on end-phenotype, making them crucial techniques for studying plant quality and environmental responses. While numerous relevant articles have been published, a comprehensive summary is currently lacking. This review aimed to understand the global and longitudinal research trends of transcriptomics and metabolomics in plant quality and environmental response (TMPQE). Utilizing bibliometric methods, we presented a comprehensive science mapping of the social structure, conceptual framework, and intellectual foundation of TMPQE. We uncovered that TMPQE research has been categorized into three distinct stages since 2020. A citation analysis of the 29 most cited articles, coupled with a content analysis of recent works (2020–2023), highlight five potential research streams in plant quality and environmental responses: (1) biosynthetic pathways, (2) abiotic stress, (3) biotic stress, (4) development and ripening, and (5) methodologies and tools. Current trends and future directions are shaped by technological advancements, species diversity, evolving research themes, and an environmental ecology focus. Overall, this review provides a novel and comprehensive perspective to understand the longitudinal trend on TMPQE.

## 1. Introduction

Since the launch of the Human Genome Project [1] in 1990, an array of genomic initiatives, such as the Earth BioGenome Project [2] and the Crop Genome Project [3], have emerged in the past 30 years, and these research data are maximally shared. Since 2000, the whole genome sequences of *Arabidopsis thaliana* [4] and rice [5] have been published. The focus of plant research has shifted from structural genomes to functional genomes, thereby bringing plant science into the post-genomic stage. With the rapid development of sequencing technologies, large amounts of omic datasets have been generated; these technologies include next-generation sequencing (NGS) [6], single-molecule real-time sequencing (SMRT) [7], chromatography and mass spectrometry technologies [8], and bioinformatic tools. Omics refers to the study of the overall DNA or genes (genome and transcriptome), proteins (proteome), and metabolites (metabolomics), and omics affect and regulate one another. However, single omics is one-sided in the study of complex plant systems; thus, multi-omic analysis must be used to integrate transcriptome and metabolome techniques for research [9].

Nonetheless, the growth habits of plants and their interactions with the environment are very complex and diverse. Even for similar species, the phenotypes of various ecotypes obviously differ due to different environments, which is one of the huge challenges in studying plant science [10]. The multi-omic technology of plants is crucial for studying their quality and relationship with environmental responses. The transcriptome encompasses the full range of RNA molecules, such as messenger RNA (mRNA), ribosomal RNA (rRNA), transfer RNA (tRNA), and non-coding RNAs that act as a crucial bridge between an organism’s genetic blueprint and the manifestation of its biological functions [11]. As the final products of various biochemical processes catalysed by enzymes, metabolites provide useful molecular insights for the biochemistry of organisms at a given time [8]. They are closely related to plant quality. Primary metabolites affect plant growth and development, and secondary metabolites could help plants resist environmental stress. Metabolomics aims to provide a global picture of all small-molecule metabolites in cells and biological fluids without observational bias that is inherent in a more concentrated metabolic institute [12]. Transcriptomics and metabolomics are complementary approaches that together provide a more comprehensive understanding of plant biology. While transcriptomics focuses on the genes and their expression, metabolomics looks at the end products of these genes and their interactions in metabolic pathways. Integrated metabolomics and transcriptomics rapidly pinpoint functional genes associated with metabolism, mitigating false positives and refining the scope for subsequent validation. This integrated approach also enables the analysis of material variance mechanisms across multiple molecular levels and from various perspectives. Meanwhile, the results of the two omics can be mutually verified in reverse genetics to analyse the mechanism of the target gene control phenotype [13]. Many studies on quality and environmental response were conducted in *Arabidopsis* [14], tomato [15], grape [16], and *Cistanche deserticola* [17] through integrated transcriptomics and metabolomics. However, there is a lack of integration and summary of the existing articles in the field. Therefore, we aim to create a scientific map that explores the current research hotspots and future directions of transcriptomics and metabolomics in relation to plant quality and environmental responses (TMPQE). This approach will help to elucidate the research focal points and trends of the past decades, synthesize the existing body of research, identify the scientific issues that require attention, delineate the characteristics and focus of contemporary investigations, and recognize the scholars and publications that are influential in the domain. This will provide insights into the trajectory of ongoing research and future research directions.

Science mapping aims to display the structural and dynamic aspects of scientific research. Three structures of knowledge-discovering hidden patterns include conceptual structure, intellectual structure, and social structure. Bibliometric analysis is a helpful method that includes statistical analysis of published articles and their citations to measure their effect and reduce prejudice [18]. The aggregate bibliographic data provided by other scientists working in the field enable researchers to ground their research in a well-established knowledge base [19]. In addition, this analysis could be used to describe development trends in academic research, explore current research priorities and hotspots, and predict future research priorities and achievements [20]. Combining co-citation analysis and content analysis could effectively organise the research streams in the research field and deepen the understanding of this field [21]. Here, a bibliometric analysis of the global studies on TMPQE was conducted to reveal the science mapping of TMPQE and identify academic cooperation networks, influential authors and journals, the evolution of research, and emerging trends. We anticipate that this review will be beneficial not only for researchers engaged in this field, but also for students and researchers with a broader interest in TMPQE.

## 2. Methodology

### 2.1. Data Collection and Filtering

**Figure 1:**
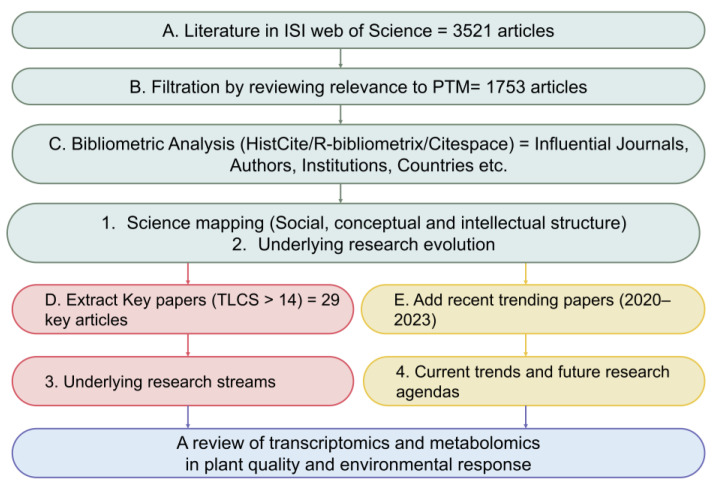
Research methodology. TLC refers to total local citations.

Input data were collected from the Web of Science (WoS) core database; the database search equations are listed in Table S1. The filtered research strategy (Figure 1) used in this review was conducted according to the process of Dhanavanth [19]. A total of 3521 articles satisfied the search criteria for bibliometric studies on TMPQE from 1994 to 2020. After duplicate data were deleted on CiteSpace 5.6.R3 software [22], the final bibliometric database included 1753 articles, 9764 authors, 217 journals, 70,180 citations, and 4669 words. Articles from the last three years (2020–2023) indicate current trends and future research agendas.

### 2.2. Bibliometric Analysis

The analysis of bibliometric networks has advanced rapidly in recent years. This analysis could reveal relationships by establishing nodes and linking networks, including co-citation, bibliographic coupling, co-authorship, and co-occurrence. Co-citation analysis [23] uses co-citation counts to construct measures of similarity between documents, authors, or journals; it is the most used and validated bibliometric method. Bibliographic coupling [24] connects documents, authors, or journals on the basis of the number of shared references. Thus, it could be used for new publications that are not cited yet, emerging fields, and small subfields. Co-author analysis [25] examines the scientists’ social networks created by collaborating on scientific articles. Co-occurrence analysis [26] is a content analysis technique that uses the words in documents to establish relationships and build a conceptual structure of the domain.

In this study, HistCite software [27] and Bibliometrix (R package) [28] were used to calculate the total number of publications (TPs), total local citation score (TLCS), average local citations received per year (TLCS/t), local citation score excluding self-citations (TLCSx), h-index, total local cited references (TLCRs), total local citation score (TLCS), and total global citation score (TGCS). These values were used to evaluate influential journals, authors, countries, and institutions. CiteSpace 5.6.R3 software was used to analyse and visualise the co-citation network of author–country–institution and the co-occurrence network of keywords. Bibliometrix (R package) was used to build up the bibliographic coupling network of the articles.

### 2.3. Citation Mapping and Content Analysis

The ‘Graph Maker’ tool in HistCite was used to filter the important core articles, visualise the result and make the database simple and usable. Filtering the TLCS value to >14 yielded 29 articles, which could be regarded as the most cited ones amongst the research on TMPQE application. This technique enabled the identification of the broader group of articles published and helped uncover groups of themes or research streams.

### 2.4. Burst Detection

Burst detection [29] is a primitive computing technique that detects sharp changes in events. Incident bursts indicate a dramatic increase in events. For example, a keyword is defined as a burst detection term if its frequency of occurrence in the last year shows a higher growth rate than other keywords. Burst detection can be twofold: (a) the strength and duration of the burst and (b) burst keywords as a key direction in development trends. CiteSpace 5.6.R3 was used for burst detection and visualization.

## 3. Results and Discussion

### 3.1. Three Stages in the Development of TMPQE

**Figure 2:**
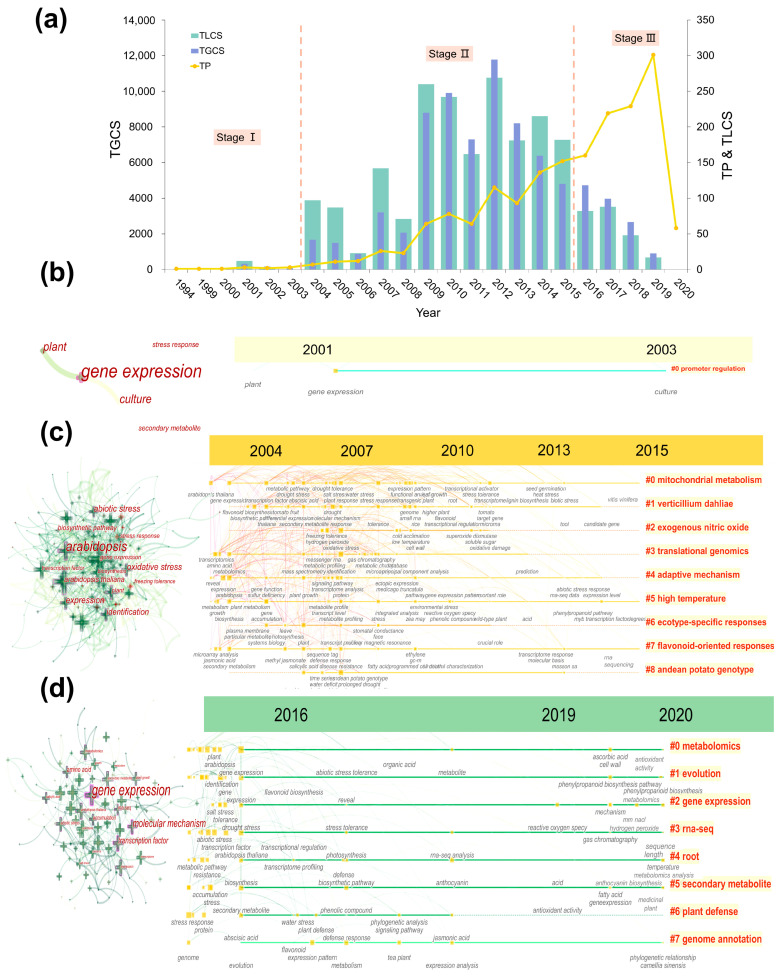
(**a**) Timeline of publications in research on application of transcriptomics and metabolomics in plant quality and environmental response (TMPQE) during 1994–2020. TPs: total number of publications; TLCS: total local citation score; TGCS: total global citation score. (**b**) Major keywords network and timeline view of co-occurrence during 1994–2003. (**c**) Major keywords network and timeline view of co-occurrence during 2004–2015. (**d**) Major keywords network and timeline view of co-occurrence during 2016–2020.

A basic descriptive analysis of yearly aggregated-level publications and citations of approximately 1753 articles from 1994 to 2020 was conducted (Figure 2a). The horizontal axis showed the year of publication, while the primary axis and the secondary axis displayed the total number of articles published (TPs), total local citation score (TLCS), and total global citation score (TGCS). Total local citations score (TLCS) refers to the number of citations received by the articles published in that year from the sample of 1753 articles, and total global citations score (TGCS) is the total number of citations received by the articles published in that year from the entire WoS database. The number of articles has been steadily increasing since 2004 and has risen sharply since 2016. Therefore, the development of TMPQE can be roughly divided into three stages in accordance with the TPs. The specific methodologies used in the bibliometric analysis to categorize TMPQE research into distinct stages are HistCite software and Bibliometrix (R package), which were used to calculate the TPs: stage I, 1994–2003; stage II, 2004–2015; and stage III, 2016–2020. Stage I is considered the embryonic period, when fewer than five papers were published each year. Stage II is called the development period, which is when the TP curve slightly fluctuates. At this stage, year 2012 received the highest TLCS and TGCS.

Stage III is called the outbreak period. Given that a certain amount of time is needed to accumulate the influence of an article after it is published, the published articles in stage III have not received many citations compared with those in stage II. This finding demonstrates that TMPQE has recently attracted attention. This attention may increase in the next few years because of the implementation of worldwide genome projects [2,30,31] and the rapid development of sequencing technologies [32,33].

Co-word analysis was conducted on the topics of each stage to further study the difference amongst the three stages in detail and clarify the thematic evolution of TMPQE research. The co-occurrence of the Keywords Plus network and timeline in the three stages represented the evolution of themes. These clusters were labelled by index terms from their own citers. Table S2 provides a list of the top three clusters in stages II and III. Figure 2b displays that in stage I (1994–2003), the main topics were gene expression, plants, culture, secondary metabolism, and stress response. The three largest clusters, namely, cluster #0 (Arabidopsis leaves), cluster #1 (maize pulvini), and cluster #2 (specific function), are summarised in Table S2. This stage centred on basic plant mechanisms, including gene expression and stress responses, with a focus on model species, thereby laying the foundation for future studies.

Figure 2c indicates that in stage II (2004–2015), the principal topics were *Arabidopsis*, gene expression, oxidative stress, biosynthetic pathway, and abiotic stress. The network was divided into nine clusters. The three largest clusters, namely, cluster #0 (mitochondrial metabolism), cluster #1 (*Verticillium dahliae*), and cluster #5 (high temperature), are summarised in Table S2. The largest cluster, cluster #0, had 31 members, a silhouette value of 0.692, and a mean year of 2008. The second largest cluster, cluster #1, had 28 members, a silhouette value of 0.589, and a mean year of 2008. The third largest cluster, cluster #5, had 22 members, a silhouette value of 0.685, and a mean year of 2007. The most active citer in these three clusters was Behnke, Katja (2010), who wrote ‘*Rnai-mediated suppression of isoprene emission in poplar transiently impacts phenolic metabolism under high temperature and high light intensities: a transcriptomic and metabolomic*’ [34]. This stage saw a focus on specialized areas like oxidative and abiotic stress, with key studies on plant metabolism and stress responses, particularly under high-temperature conditions.

Figure 2d indicates that in stage III (2016–2020), the major topics were gene expression, molecular mechanism, transcription factor, and amino acid. The network was divided into eight clusters. The three largest clusters, namely, cluster #0 (metabolome analyses), cluster #1 (candidate gene and cluster), and cluster #3 (endogenous auxin level, with a mean year of 2017), are summarised in Table S2. The largest cluster, cluster #0, had 17 members and a silhouette value of 0.839. The most active citer was Tripathi, Sandhya (2020), who wrote ‘*Berry transcriptome: insights into a novel resource to understand development dependent secondary metabolism in withania somnifera (ashwagandha)*’ [35]. The second largest cluster, cluster #1, had 16 members and a silhouette value of 0.848. The most active citer was Wu, Peipei (2020), who wrote ‘*Berry transcriptome: insights into a novel resource to understand development dependent secondary metaboli*’ [36].The third largest cluster, cluster #3, had 14 members and a silhouette value of 0.82. The most active citer was the same as with cluster #0. During stage III, the major topics are key to plant development and stress responses. The focus on metabolome analyses and endogenous auxin levels suggests a move towards a systems biology approach, highlighting the integration of various biological aspects to understand complex plant processes. This trend is expected to continue, with potential future growth in the application of advanced sequencing technologies and the exploration of plant–environment interactions.

### 3.2. Social Structure

#### 3.2.1. Leading Journals

**Figure 3:**
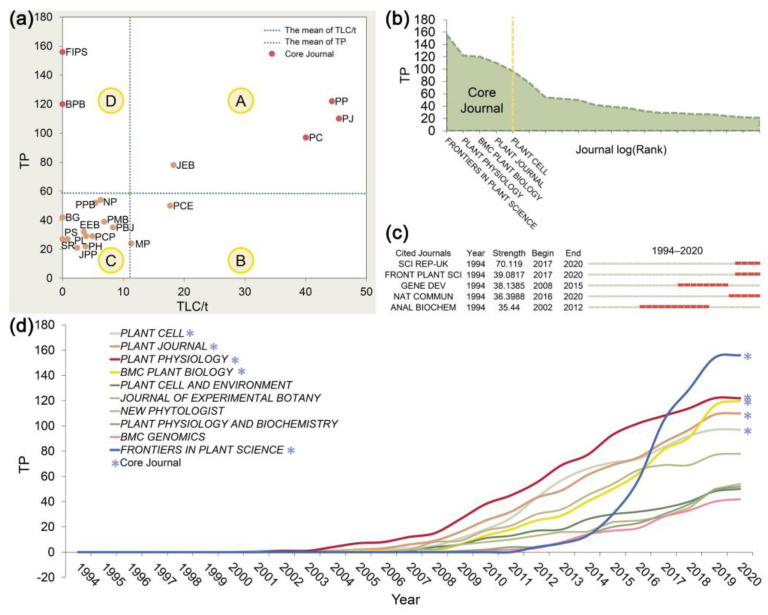
Leading journals in TMPQE during 1994–2020. (**a**) Journal focus and impact on TMPQE research. A four-quadrant scatter graph on the basis of TP (as a proxy for focus on TMPQE) and TLCS/t (as a proxy for impact): A, high focus on TMPQE and high impact; B, low focus on TMPQE but high impact; C, low focus on TMPQE and low impact; D, high focus on TMPQE but low impact. (**b**) Journal clustering through Bradford’s law. Bradford’s law can be used to identify core journal in discipline and eventually to focus the analysis on the core zone documents. (**c**) Top five journals with strongest burst from 1994 to 2020. (**d**) Journal dynamics. Journal number of publications per year. TPs: total number of publications, TLC/t: average local citations received per year.

A total of 1753 articles were published in 216 journals. The top 10 leading journals in TMPQE research are presented in Table S3. The journals were ranked on the basis of two parameters, namely, the TP related to TMPQE and the TLCS/t. Amongst the top 10 journals, *PLANT PHYSIOLOGY* (PP), *THE PLANT JOURNAL* (PJ), and *THE PLANT CELL* (PC) were the most productive journals. Even though *FRONTIERS IN PLANT SCIENCE* (FIPS) and *BMC PLANT BIOLOGY* (BPB) journals were in the top 10 of TP ranking, they did not take any place in the list when ranked by TLCS/t. Both journals are low TMPQE-impact journals (Figure 3a). In addition, FIPS is a relatively new journal inaugurated in 2010; thus, some time is needed for the journal to attract attention and make an impact. The dynamic curve (Figure 3d) of the journals demonstrated that FIPS has published a significant number of TMPQE-related articles between 2014 and 2020.

Only PP, PJ, PC, and *JOURNAL OF EXPERIMENTAL BOTANY* (JEB) belonged to quadrant A (high focus on TMPQE and high impact) (Figure 3a). The dynamic curve (Figure 3d) of the journals also displayed that the TPs by these four journals has been at a relatively high level, and their average TPs since 1994 are higher than the average (total average: 20.5, PP: 37.7, PJ: 29.5, PC: 27.9, JEB: 21.4). *THE PLANT CELL AND ENVIRONMENT* (PCE) and *MOLECULAR PLANT* (MP) belonged to quadrant B. Similar to Table S3, FIPS and BPB belonged to quadrant D, with higher-than-average TPs but lower-than-average TLCS/t.

Figure 3c shows five journals with burst detection from 1994 to 2020. Amongst these journals, *SCIENTIFIC REPORTS* (SR), FIPS, and *NATURE COMMUNICATIONS* (NC) exhibited the highest strength (SR: 70.119, FIPS: 39.0817 and NC: 36.3988). Bradford’s law [37] was used to identity the core journals of TMPQE. Figure 3b reveals the five core journals, namely, FIPS, PP, BPB, PJ, and PC, which all published approximately 51.8% of the articles of the entire TMPQE collection.

#### 3.2.2. Influential Authors

**Figure 4:**
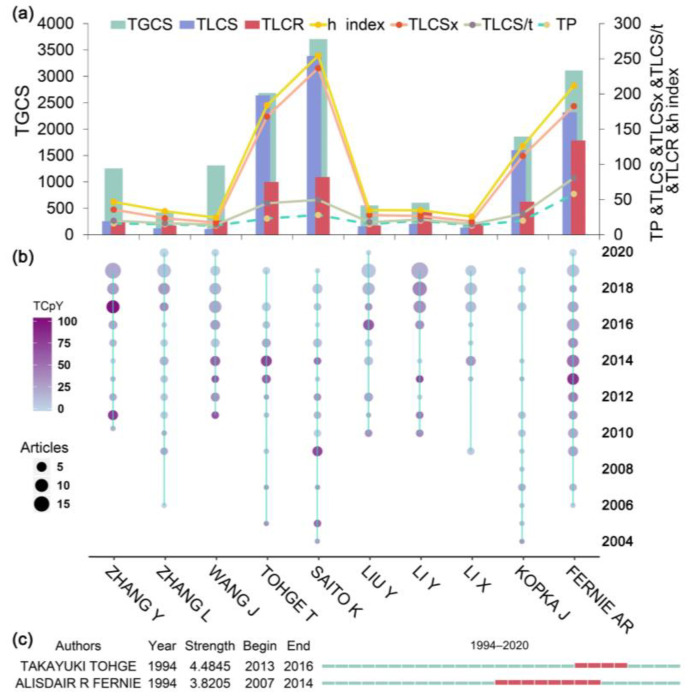
Influential authors of TMPQE during 1994–2020. (**a**) The top 10 productive authors in the publication of TMPQE during 1994–2020.TLCRs: the total number of citations in an article’s reference list to other articles within the collection. TLCSx: the total local citation scores that do not include self-citation. The h-index: the author’s number of published articles (*h*), each of which has been cited in other papers at least *h* time. (**b**) Author’s production over time. The line represents an author’s timeline. The bubble size is proportional to the number of articles. The colour intensity is proportional to the total citations per year. (**c**) Authors with strongest burst from 1994 to 2020. TPs: total number of publications, TLCS/t: average local citations received per year, TLCSx: local citation score excluding self-citations, h-index: an unbiased indicator to assess the performance of scientific outputs according to quantity and quality aspects, TLCRs: total local cited references, TLCS: total local citation score, TGCS: total global citation score.

Figure 4a presents the top 10 productive and most influential authors in the TMPQE field. The top three authors were Fernie AR (Max Planck Inst Mol Pflanzenphysiol, Germany), Saito K (Chiba University, Japan), and Tohge T (Max Planck Inst Mol Plant Physiol, Germany), who all had high TPs and influence, including TLCS, TGCS, TLCSx, TLCRs, TLCS/t, and h-index [38]. The author’s production over time plot (Figure 4b) demonstrated that the three authors mentioned above have been publishing high-impact articles from 2004 (except Fernie AR from 2016) to the present. The authors’ burst detection result (Figure 4c) was consistent with that in Figure 4b, which shows that Tohge T exhibited the highest strength of 4.4845 from 2013 to 2016 and Fernie AR exhibited the strength of 3.8205 from 2007 to 2014.

#### 3.2.3. Country Performance

**Figure 5:**
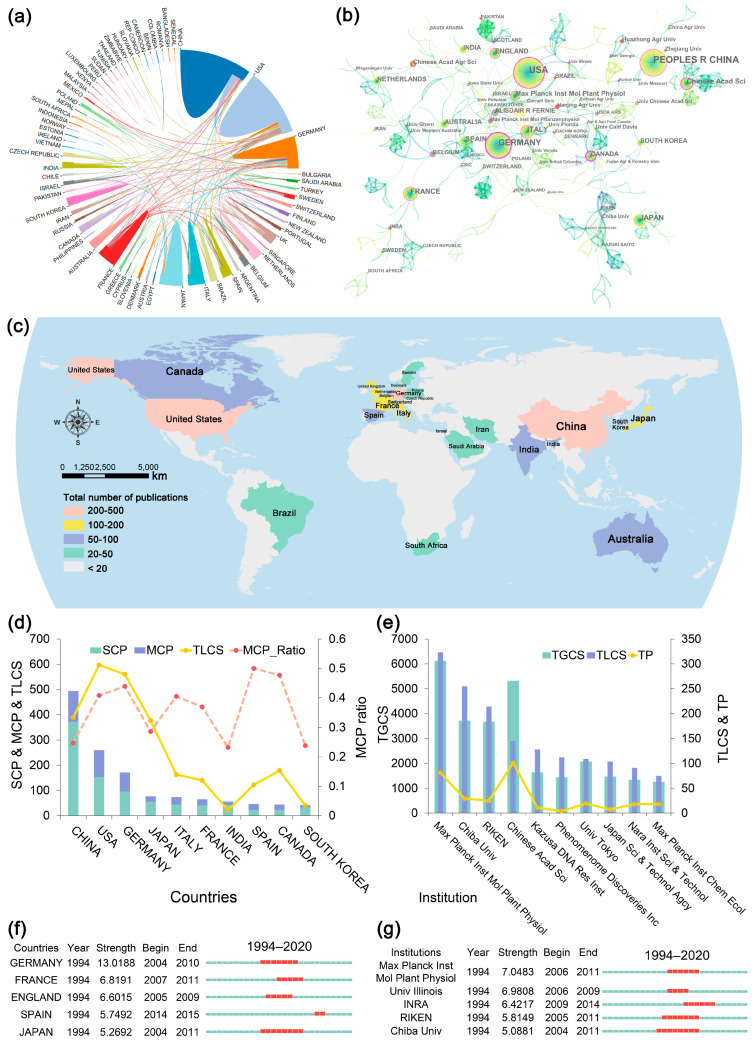
Social structure of TMPQE during 1994–2020. (**a**) Academic cooperation network between countries/regions. (**b**) International collaborations among the most productive institutions, countries, and authors. (**c**) Geographical distribution map. (**d**) Top 10 corresponding author’s countries. (**e**) Top 10 productive institutions. (**f**) Top 5 countries with strongest burst from 1994 to 2020. (**g**) Top 5 institutions with strongest burst from 1994 to 2020. TPs: total number of publications, TLCS: total local citation score, TGCS: total global citation score, SCP: single country publication, MCP: multiple countries publication, MCP ratio: measure the international collaboration intensity of a country.

A total of 72 countries contributed to articles regarding TMPQE from 1994 to 2020. The map of the TPs in each country (Figure 5c) drawn using ArcGIS software indicated that the countries with more than 200 articles were China (550), the United States (420), and Germany (275), whilst the countries with more than 100 articles included France (121), Italy (113), Japan (110), and the United Kingdom (105). Figure 5d indicates that except for China, India, and South Korea, the TLCS of the other top 10 countries are higher than their TPs, especially for the United States, Germany, and Japan. Figure 5d also demonstrates that the countries with high international collaboration intensity were Spain, Canada, and Germany. The countries’ burst detection result (Figure 5f) revealed that Germany and Japan began in 2004.

#### 3.2.4. Centres of Excellence

Figure 5e indicates that the TLCSs of the top 10 institutions (except Chinese Academic of Science) were larger than the TGCSs, indicating that these institutions were more influential and pioneering in the TMPQE field. The top three most influential institutions were Max Planck Institute of Molecular Plant Physiology (Germany), Chiba University (Japan), and RIKEN (Japan). The institutions’ burst detection result (Figure 5g) demonstrated that these institutions began in 2004, which was consistent with the results in countries and authors.

#### 3.2.5. Academic Collaboration

The circle plot of cooperation between countries (Figure 5a) illustrated the top three countries (China, the United States, and Germany) with the highest TPs worked in close cooperation with each other. The network diagram (Figure 5b) of the country–author–institution relationship showed the international collaboration circle in TMPQE, with Germany, Japan, and China as the core. The main collaboration countries in the Germany-centric cooperation circle were the United States, Italy, and Spain. The main cooperation institution was the Max Planck Institute of Molecular Plant Physiology, and the core authors were Fernie AR and Tohge T. The collaboration circle centred on Japan was gathered together; the main institutions were Chiba University and RIKEN, and the core author was Saito K of Chiba University. The main institutions in the cooperation circle centred on China included the Chinese Academy of Sciences, China Agricultural University, Huazhong Agricultural University and Zhejiang University. The results of the burst detection by countries, institutions, and authors demonstrated that in stage II (2004–2015), Fernie AR and Tohge T from the Max Planck Institute of Molecular Plant Physiology in Germany and Saito K from Chiba University and RIKEN institution in Japan paid attention to and influenced the TPMQE research starting from 2004. The institutions in China and the United States have contributed numerous articles in the TMPQE field in recent years.

### 3.3. Conceptual Structure

#### 3.3.1. Subject Category Analysis

**Figure 6:**
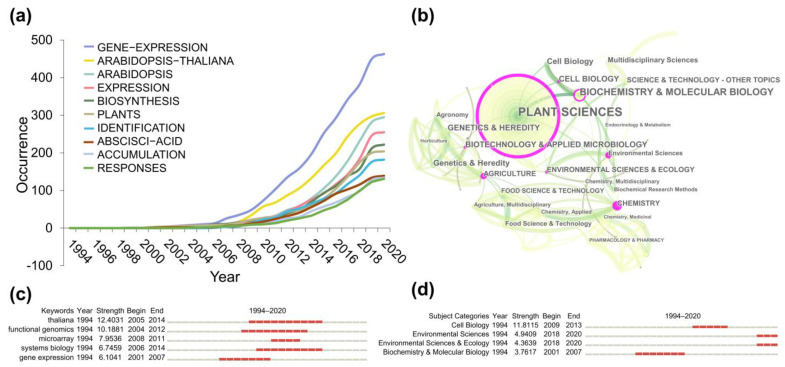
Conceptual structure of TMPQE during 1994–2020. (**a**) Keywords number of occurrences per year. (**b**) Major disciplines network. (**c**) Top 5 keywords with strongest burst from 1994 to 2020. (**d**) Subject categories with strongest burst from 1994 to 2020.

All of the 1753 publications related to TMPQE were divided into 39 categories in the WoS core collection database. The subject category network (Figure 6b) showed the co-citation relationship among various disciplines. The top 3 frequent subjects are plant science (1443), biochemistry, and molecular biology (309). The high-intermediate centrality between the nodes with the thick purple ring represents a great potential for scientific transformation, and the values identify potential boundary crossings that may lead to transformation discovery. The subject category’s burst detection result (Figure 6d) showed that cell biology possessed the highest burst strength of 11.8115 from 2009 to 2013. Meanwhile, environmental sciences and ecology possessed burst strengths of 4.9409 and 4.3639 since 2018, respectively, indicating that these two subjects have been the novel popular disciplines in recent years.

#### 3.3.2. Hot Topics

Keywords Plus [39] contains words or phrases that appear frequently in the titles of an article’s references and not necessarily in the title of the article or as author keywords. It is generated by an automatic computer algorithm. Thus, Keywords Plus is as effective as author keywords in terms of bibliometric analysis investigating the knowledge structure of scientific fields. The dynamic curve of Keywords Plus (Figure 6a) showing gene expression and *Arabidopsis thaliana* has always been the hot words in TMPQE research. The subject category’s burst detection result (Figure 6c) demonstrated that *A. thaliana* had the highest burst strength of 12.4031 from 2005 to 2014, followed by functional genomics at 10.1881 from 2004 to 2012. A microarray showed a high burst strength of 7.936 from 2008 to 2016, indicating that gene microarray occupied a dominant position in TMPQE research technology before the development of RNA-seq.

Co-word analysis is more intuitive than co-citation analysis and bibliographic coupling analysis. In science mapping, a network graph is used to represent co-occurrences amongst bibliographic metadata. In this study, a network of conceptual structure (Figure S1a) obtained via co-occurrence analysis of Keywords Plus revealed hot topics, including gene expression, *Arabidopsis*, biosynthesis, transcription factor, identification, accumulation, and stress in the TPMQE field. Figure S1b shows CiteSpace’s timeline visualization analysis of the TPMQE study through 2020. The keywords are shown in time sequence on the horizontal axis. They are also visualised by a broad topic on the vertical axis and expressed by clusters of related research [40]. The largest cluster was placed at the top of the diagram. Each circle represented the cited article. When the height is quoted, the circle diameter increases [41]. The network was divided into 14 co-occurrence clusters. These clusters were labelled by index terms from their own citers. The five largest clusters included cluster #0 (*Arabidopsis* leaves), cluster #1 (maize pulvini), cluster #2 (specific function), cluster #3 (*Panicum virgatum*) and cluster #4 (secondary metabolite), as shown in Table S2. The silhouette values amongst the top five clusters were in the range of 0.750–0.858, indicating high homogeneity and meaningful cluster results [42]. The largest cluster (#0) showed 28 members with a silhouette value of 0.868; it is labelled as *Arabidopsis* leaf by log-likelihood ratio. The most active citer in cluster #0 was Liu, Xiaoli (2011), who wrote ‘*Metabolomic study on the halophyte suaeda salsa in the yellow river delta*’ [43]. Furthermore, cluster #0 was one of the oldest clusters with a mean year of 2008.

#### 3.3.3. Theoretical Basis

The highly cited articles in the TMPQE dataset are extremely considerable and pioneering. They often represent basic theories and methodologies in the TMPQE field. Table S6 shows the top 10 cited articles in TMPQE research. These articles were all related to methodology or software development. They could be divided into three classes: (a) In the theoretical algorithm, article #1 [44] explored qPCR and 2^−ΔΔCt^ method to analysis relative gene expression data, and article #2 [45] was about the algorithm of controlling the false discovery rate of multiple testing. (b) In the development of bioinformatic tools and software, article #3 [46] discussed MapMan, a user-driven tool to display genomic datasets onto diagrams of metabolic pathways and other biological processes. Article #4 [47] described the assembly of full-length transcriptomes without a reference genome using Trinity. Article #6 [48] depicted Blast2GO, a universal tool for annotation, visualization and analysis in functional genomic research. Article #10 [49] reported on differential expression and gene analysis of RNA-seq through TopHat and Cufflinks. (c) In terms of establishing experimental methods and protocol, article #5 [50] explored Floral dip, a simplified method for agrobacterium-mediated transformation of *A. thaliana*. Article #7 [51] introduced a protocol for metabolite analysis on the basis of gas chromatography–mass spectrometry (GC–MS). Article #8 [52] showed the mapping and quantifying mammalian transcriptomes by RNA-Seq. Article #9 [53] presented a rapid and sensitive method for the quantitation of microgram quantities of protein utilizing the principle of protein–dye binding. In summary, these 10 articles provided a theoretical basis for TMPQE research in three aspects: theoretical algorithm, experimental protocol, and bioinformatic software development. Furthermore, they mainly originated from the United States, Germany, and Israel.

### 3.4. Intellectual Structure

Intellectual structure discusses how the work of an author influences a given scientific community [54,55]. A three-field plot (Figure S3) indicated the relationship amongst top Keywords Plus networks, top authors, and top journals. In TMPQE research, Femie AR, Tohget T, and Saito K have made outstanding contributions in plants, gene expression, *A. thaliana*, and biosynthesis research and published articles in *PLANT PHYSIOLOGY*, *THE PLANT JOURNAL*, *THE PLANT CELL,* and *FRONTIERS IN PLANT SCIENCE.*

### 3.5. Research Streams and Future Research Directions

**Figure 7:**
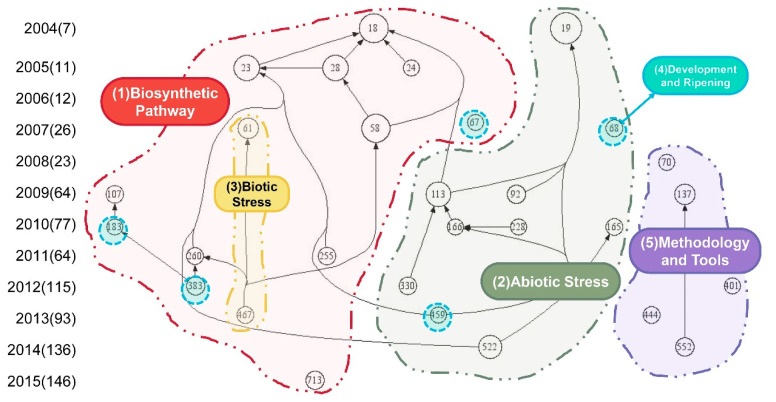
Citation mapping of the most influential articles in TMP research. (The numbers in the figure represent the Node Numbe of the 29 core articles, the corresponding articles can be found in Table S7)

As shown in Figure 7, a citation map was created using co-citation analysis to demonstrate the evolution of TMPQE research over time. Filtering the TLC value to ≥ 14 resulted in 29 articles, which were regarded as the most cited articles in the TMPQE literature. The basic assumption of co-citation analysis is that the more two items are cited, the more their content is related [56]. Thus, citation map is beneficial for identifying research streams in the research field. Content analysis was used to identify and record the relatively objective (or at least inter-subject) characteristic of a message [19,21]. While content analysis involving multiple researchers can enhance the reliability of results, it is important to recognize that citation counts have limitations, such as potentially favouring highly cited articles that may not always reflect the most influential or innovative research, overlooking recent impactful work due to the time needed for citations to accumulate, and sometimes reflecting increased attention due to controversy rather than methodological rigor. As a result of the coupling content and co-citation analysis of 29 core articles (Table S7), five distinctive but interrelated research streams on TMPQE were identified: (1) biosynthetic pathway (red area), (2) abiotic stress (green area), (3) biotic stress (yellow area), (4) development and ripening (cyan area), and (5) methodology and tools (purple area).The key theories, methods, and findings of the article are briefly discussed in Figure 7 in the context of their respective research streams and sub-streams. In addition, combining 29 core articles and new TMPQE-related papers published in the last three years demonstrates how these streams developed, and their relevance for future research is indicated.

#### 3.5.1. Biosynthetic Pathway

The first research streams contained 17 of the 29 core articles that studied the plant biosynthetic pathways through transcriptomic and metabolomic techniques. This research flow was classified in accordance with the following experimental methods: (a) Some research compared the differences in transcription and metabolic profiles under different variables (including different environmental treatments, tissues, or growth times) from the overall level to explain the molecular mechanisms of variables on plant transcription and metabolite biosynthesis [14,57,58,59,60]. The study of transcription profiles included comparing gene expression levels and identifying single-nucleotide polymorphisms (SNPs) and alternative splicing isoforms [61,62]. (b) Some studies identified the function of unknown genes or transcription factors through over-expression, ectopic expression, or knockout of specified genes or transcription factors to prove their molecular mechanism in a metabolite biosynthetic pathway [63,64,65]. (c) Some studies explored key gene clusters or transcription factors that regulate a certain metabolite biosynthetic pathway such as anthocyanin, flavonoid, and glucosinolate biosynthesis, through targeted metabolite measurement, overexpression, and knockout [66,67,68]. One article has examined the biosynthetic pathways of compounds with special value, including tanshinone [62].

The earliest article in this research stream came from Chiba University (Japan) and was published in *PROCEEDINGS OF THE NATIONAL ACADEMY OF SCIENCES OF THE UNITED STATES OF AMERICA* in 2004 [14]. This article integrated transcriptomics and metabolomics to elucidate the global response to nutritional stress of *A. thaliana* and the gene-metabolite network in primary and secondary metabolisms. These metabolisms which could identify gene functions and subsequently improve the production of useful compounds in plants and provide a paradigm for later research. Article 255 [65] explored gene–anthocyanin content–protein interactions. Besides transcriptomic and metabolomic techniques, yeast two-hybrid assay, CHIP analysis, BiFC analysis, and CoIP analysis were used to reveal a direct link between the transition to flowering and secondary metabolism and provide a potential target for manipulation of anthocyanin and flavonol content in plants. This article provided detailed research methods for the subsequent integration of transcriptome and metabolome with other protein interactions in studying the plant biosynthetic pathways. In 2015, the Institute of Medicinal Plant Development, Chinese Academy of Medical Sciences, published article 713 [62]. This article described the full-length transcriptome sequence of *Salvia miltiorrhiza* obtained by NGS and SMRT sequencing and examined long non-coding RNA (lncRNA) and splice variants. It also studied the molecular mechanism of tanshinone biosynthesis in different tissues at the transcription-metabolism level.

#### 3.5.2. Abiotic Stress

The research stream encompassed 10 articles that explored the interaction between abiotic stress and plant quality at both the transcriptional and metabolic levels. Section 3.5.2 and Section 3.5.3 could be integrated to investigate the influence of the environment on plant quality. In this research stream, the key research contents involved the influence and response of temperature treatment [69], water treatment [68], salt stress [70], light and ultraviolet-B radiation [71], and oxidative stress [72] on plant quality. Moreover, water treatment included drought stress [73,74], dehydration [75], and osmotic stress [76]. On the basis of different research objects, this research stream could be divided into two categories: to determine the impact of environment on plant metabolism, and to study a metabolite with a special function that could change the response of plants to the environment. Thus, the technology of this research stream was more focused on metabolomic analysis. In the study of the effect of abiotic stress on plant metabolism, using non-targeted metabolomics could observe the dynamic changes in the plant’s overall metabolic network [69,75]. In addition, targeted metabolomics can reveal the characteristics of specific metabolites, such as flavonoids, organic acids, and amino acids [68,70].

In 2004, the earliest article, which was article 19 [69], in this research stream was published in PP by the Max Planck Institute of Molecular Plant Physiology in Germany. This article provided new insights into the mechanisms by which plants adapt to heat stress at the metabolite level. Articles 92 [70] and 330 [74] explored the effects of abiotic stress on transcription and metabolism amongst various plant ecotypes. Under abiotic stress, plants exhibit a molecular response with the upregulation of stress-responsive genes and the downregulation of those associated with growth, highlighting their adaptation mechanisms. Concurrently, metabolic reprogramming results in the accumulation of protective osmoprotectants and antioxidants, essential for maintaining cellular homeostasis. These findings enhance our understanding of plant stress responses, informing the development of stress-tolerant crop varieties and informing agricultural practices to optimize plant growth under environmental challenges.

#### 3.5.3. Biotic Stress

The third research stream was the biological stress included in the first research stream. The main research topic was the mechanism of transcription factor regulating glucosinolate biosynthesis under insect induction. Article 61 [67] identified new regulatory genes for the glucosinolate biosynthesis pathway by co-transformation assay. It revealed that the R2R3-MYB transcription factor HAG1/MYB28 regulates methionine-derived glucosinolate biosynthesis in *A. thaliana*, thereby promoting the plant’s long-lasting resistance to herbivores. In article 467 [77], gene microarray, UPLC-QTOF, qPCR, CHIP, and yeast two-hybrid analysis were used to reveal that *A. thaliana* basic helix-loop-helix transcription factors MYC2, MYC3, and MYC4 regulate glucosinolate biosynthesis, and insect performance and feeding behaviour. The degradation products of glucosinolate have a certain biological activity and special smell, which could give a deterrent effect on certain insects and herbivores [78].

#### 3.5.4. Development and Ripening

The fourth research stream was scattered in Section 3.5.1 and Section 3.5.2, and the main research topic was the characteristics of transcription and metabolism during plant development and ripening, in which both often change in phenotype, especially in colour. Anthocyanin is a class of flavonoids derived from phenylalanine and widely present in the cytosol of flowers, fruits, stems, leaves, and root organs, making it appear in different colours, from red to purple to blue [79]. Most articles in this research stream studied the features of anthocyanins during the development and ripening of grape [59,61,68,80]. They indicated that grape is particularly suitable for research on anthocyanins. Article 459 [60] comprehensively dissected the spatiotemporal metabolic shifts in primary, secondary and lipid metabolisms during developmental senescence in *Arabidopsis*, serving as a blueprint for the analysis of traits and conditions linking crop yield and senescence.

#### 3.5.5. Methodology and Tools

The last research stream introduced some methodologies and tools for transcriptomic and metabolomic research. Articles 137 and 552 introduced MapMan [81] and Mercator [82], which were used to visualise and compare omic data and transcriptome functional annotation in plants, respectively. Article 70 [83] proposed a set of standardised protocols for metabolomic data analysis. Article 401 [84] described in detail the steps and process of discovering and identifying lncRNA based on public databases and RNA-seq. Article 444 [85] described a methodology for co-expression network modules of plant transcripts in response to single and combined pressure.

With the development of post-genomics, a significant amount of research could be found on the integration of transcriptomics and metabolomics in plant quality and its relationship with the environment. In particular, some articles certified that integrating transcriptomic and metabolomic analyses is an effective and important method for studying plant quality across different ecotypes [70,74].

With the development of sequencing technology, gene microarray analysis was gradually converted to RNA-seq in transcriptomic analysis. In accordance with different read lengths, RNA-seq was divided into NGS and SMRT. In the 29 core articles, almost all research samples had reference genomes, and most of these were from model plants (*A. thaliana*) and cash crops (rice, wheat, and grapes). Over time, the significance of *A. thaliana* in TMPQE research has evolved from basic discovery to more applied research, including the development of stress-tolerant crop varieties for agriculture. The significance of rice in this field has grown in response to the increasing demand for food security in the face of climate change. More recent research has focused on non-model plants, especially medicinal herbs with medicinal value [62]. However, most herbs lack genomic information, and obtaining high-precision transcripts without a reference genome is a challenge. The development of SMRT makes research not limited to the study of the differences in transcript expression. Some studies have also used non-coding RNA, alternative splicing isoform and SNPs related to metabolite biosynthesis or environmental response [61].

### 3.6. Current Trends and Future Research Agendas

Bibliographic-coupled analysis explores the hidden relationships between articles and partially presents the evolving patterns of research frontiers in specific topics [86]. Bibliographic coupling network (Figure S2) with degree centralization of 0.282 revealed the current and future intellectual structures of TMPQE research.

Combining 29 core articles and new TMPQE-related papers published in the last three years discussed the current trends and future research agendas. In the last three years of research, combining other omic technologies, such as genomics, for upstream verification or single-cell technology to achieve greater refinement and precision, is becoming a trend in biosynthetic pathway. Qi TC et al. discussed the characteristics of secondary metabolites of anthocyanin accumulation [87]. Sonia Ouadi et al. compared the newly assembled clove genome with that of Eugenia caryophyllata to study genomic evolution between two genera in the Myrtaceae family, as well as the genes involved in eugenol biosynthesis [88]. Sijie Sun et al. combined single-cell transcriptional profiling to reveal that the biosynthesis of vinblastine is divided into three parts: the pathway starts in IPAP cells; intermediate steps are carried out in epidermal cells; and the last three steps of vincristine synthesis are carried out in IC cells [89]. In addition to studying some classic compounds (such as anthocyanins and flavonoids) in model species, more articles are exploring some compounds with special values in special plants, especially those with medicinal value, such as tanshinone. For abiotic stress, the investigation into the effects of atmospheric pollution, heavy metal contamination, acid rain, and other environmental pollutants on plant quality has garnered increasing attention [90,91]. This finding is consistent with the conclusion we drew through analysis in Section 3.3.1. In methodology and tools, with the explosive publication of more omics data, it is necessary to establish databases that generate searchable, accessible, interoperable, and reusable data for future use. The establishment of the grape transcriptomics and metabolomics integrated database (TransMetaDb) has set an example for other species [92]. In addition, with the progress in the field of computer science (i.e., machine learning, artificial intelligence, neural networks, etc.), research methods and depth have reached a new level. The combination of metabolomics analysis and machine learning (ML) has proven to be effective in guiding targeted improvement in fruit flavours [93]. Therefore, future TMPQE research is poised to benefit from integrating multi-omics data and leveraging artificial intelligence (AI) and ML for data analysis. Techniques like single-cell omics and in situ sequencing will provide deeper insights into plant responses, enhancing our understanding of quality and environmental interactions. These innovations will drive further advancements in plant science [94,95]. 

In summary, the results of the recent article analysis suggested four points of current trends and future directions. (1) In terms of technical and analytical transformation, with the development of sequencing technology, especially SMRT sequencing [96], TMPQE research studied the relationship between mRNA expression and phenotype and focused on lncRNAs and alternative splicing isoform. SMRT sequencing provides enhanced technical support for full-length transcriptome research. Meanwhile, establishing a comprehensive and accurate metabolite library and combining computer science has gradually become one of the hotspots in metabolomic research. (2) For changes in species, most of the previous studies were about model species or important economic crops. Nowadays, studies on non-model species, especially medicinal plants and new energy plants, are gradually increasing. (3) Research content gradually evolved from a single-omic study to a study integrating multiple and single-cell omics. Nowadays, due to the increasing and mature analytic methods of big data, more research focuses on using large numbers of omic data for overall research. For instance, weighted gene co-expression network analysis (WGCNA) is being used to explore the network regulation relationship between gene modules and phenotypes via gene-set enrichment analysis (GSEA). However, the integration of multi-omics data and the complexity of biological systems present ongoing challenges, as the stability of various omics data collections, the introduction of human interference, and the characteristics of different omics datasets all require full consideration. Therefore, when combining multi-omics data, it is crucial to eliminate noise, preprocess the data, recognize its characteristic features, employ advanced analytical tools, apply systems biology methodologies, and integrate the data with computational screening and mathematical modelling [97,98]. (4) Regarding research topics, the field of environmental ecology has been a novel and popular discipline in recent years. Building on the growing interest in environmental ecology, the field of TMPQE has seen a notable shift towards the integration of omics data, emphasizing the study of climate change impacts, the exploration of non-model plants for biodiversity, and the application of AI in data analysis, aligning with the broader goals of environmental science to tackle ecological complexities and enhance sustainable practices.

Overall, advances in sequencing and multi-omics integration have characterized TMPQE research; however, challenges remain in managing complex data and exploring biosynthetic pathways in diverse plant species. Applying findings to environmental challenges and enhancing AI and machine learning techniques are crucial next steps. These efforts will enrich our understanding of plant–environment dynamics, enhance agriculture by breeding crops with better quality and stress tolerance, and reduce the environmental impact of farming. They also promise to improve food security and public health by uncovering plant-based compounds for new therapies. Environmentally, it aids in understanding and preserving plant biodiversity for sustainable ecosystem management. Recognizing the limitations of bibliometrics, this review has mapped the overall research trends and hotspots in TMPQE, suggesting potential future trajectories. To delve deeper, we plan to employ meta-analysis and systematic reviews, aiming for a more nuanced analysis of key research areas.

## 4. Conclusions

In this study, a comprehensive bibliometric and content analysis of TMPQE research was conducted, examining 1753 articles from the WoS core database. A detailed science map of its social, conceptual, and intellectual structures, three developmental stages, four research directions, and five emerging research streams in TMPQE were identified by bibliometric and content analysis.

The conceptual and intellectual analysis highlighted the foundational role of theoretical algorithms, experimental protocols, and bioinformatic software in TMPQE research. Prominent journals, influential authors, and key international collaborations were identified, with a focus on Germany, Japan, and China as hubs of academic exchange. Additionally, the growth of TMPQE articles through the 2020s was tracked, revealing a clear progression from stage I (1994–2003) to stage III (2016–2020). Five pivotal research streams were pinpointed: biosynthetic pathways, abiotic stress, biotic stress, development and ripening, and methodology and tools. Furthermore, by integrating insights from the 29 most cited articles and recent publications (2020–2023), the study outlined the current trends and four future directions in technical and analytical transformations, species diversity, research content evolution, and environmental ecology.

This review stands as the inaugural comprehensive analysis of TMPQE research, offering a valuable reference for its temporal evolution and anticipated advancements. It recognizes the critical contributions of leading authors, institutions, and academic networks, underscoring the importance of TMPQE research in propelling plant science forward and addressing pressing global issues.

## Data Availability

All data generated or analysed during this study are included in this published article (and its Supplementary Information Files).

## Supplementary Materials

The following supporting information can be downloaded at https://www.mdpi.com/article/10.3390/metabo14050272/s1: Table S1: Web of Science search equations and number of articles per search. Table S2: The top clusters of co-occurrence in research on transcriptomics and metabolomics in plant quality and environmental response (TMPQE) during 1994–2020. Table S3: Leading journals in TMPQE research. Table S4: Journal Related Abbreviations. Table S5: Methodology related abbreviations. Table S6: Theoretical basis: top 10 cited articles in research on transcriptomics and metabolomics in plant quality and environmental response (TMPQE). Table S7: Detailed overview of the 29 most influential articles (with respect to TLC value > 14) from citation mapping. Figure S1: Conceptual structure of TMPQE during 1994–2020. (a) Major keywords network of co-occurrence. (d) Timeline view for document co-occurrence cluster. Figure S2: Bibliographic coupling analysis of the literature of TMPQE in 2016–2020. Figure S3: Sankey plot of top 20 author-top 10 keyword plus-top 10 journal in research on TMPQE.
