# Supplementary material for: A Review of Transcriptomics and Metabolomics in Plant Quality and Environmental Response: From Bibliometric Analysis to Science Mapping and Future Trends
Source: Metabolites. 2024 May 8;14(5):272. doi: 10.3390/metabo14050272 (PMC11123105; doi:10.3390/metabo14050272)
Supplement: Supplementary file 1 [file metabolites-14-00272-s001.zip › 24.04.10-Supplementary figures.pdf]

Supplementary Figures

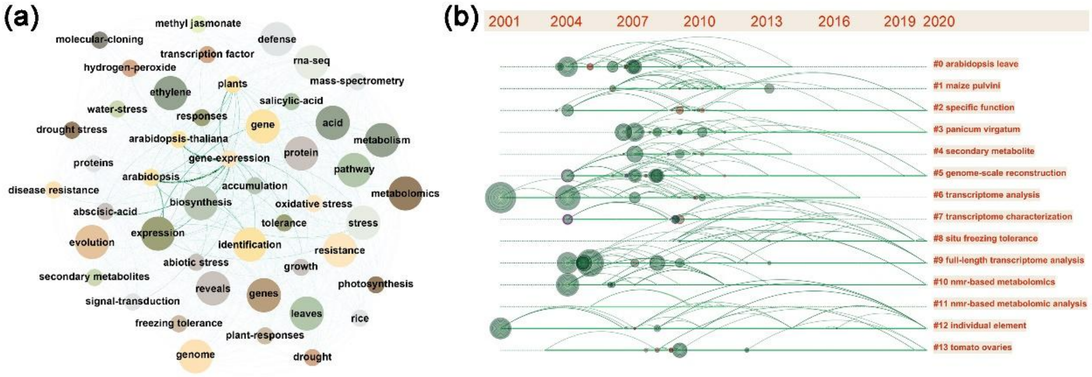

Figure S1 Conceptual structure of TMPQE during 1994–2020.
(a) Major keywords network of co-occurrence. (Nodes represent keywords, edges connect keywords that co-occur, and the width of the edges indicates the frequency of these co-occurrences)
(b) Timeline view for document co-occurrence cluster. (Each circle denotes a cited article, with its size proportional to its citation count, and these citations are chronologically ordered along the horizontal axis. The vertical axis categorizes the citations by broad topics, illustrating the interconnectedness of related research areas)

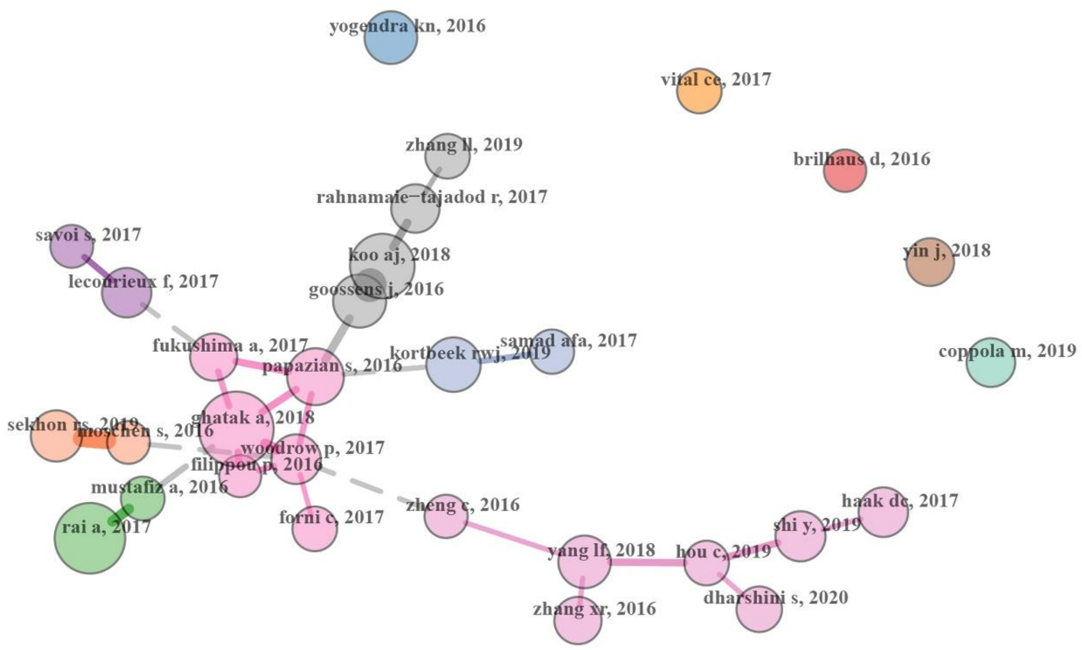

Figure S2 Bibliographic coupling analysis of the literature of TMPQE in 2016-2020（Each literature is represented as a node, with the size of the node indicating the local citation counts of the literature. The color of the node signifies the literature's cluster）

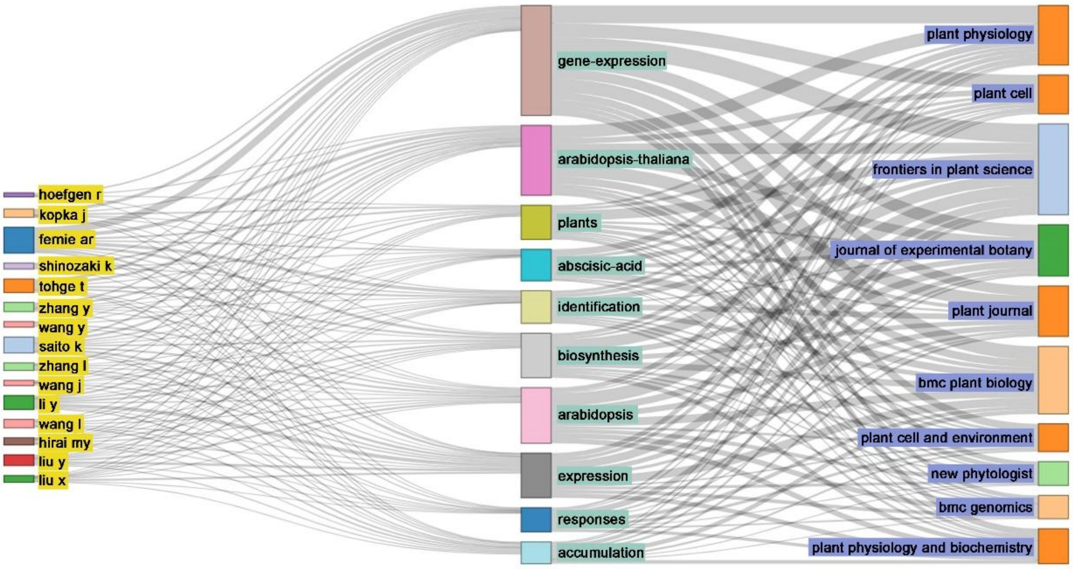

Figure S3 Sankey plot of top 20 author-top 10 keyword plus-top 10 journal in research on TMPQE.
