# Supplementary material for: A Review of Transcriptomics and Metabolomics in Plant Quality and Environmental Response: From Bibliometric Analysis to Science Mapping and Future Trends
Source: Metabolites. 2024 May 8;14(5):272. doi: 10.3390/metabo14050272 (PMC11123105; doi:10.3390/metabo14050272)
Supplement: Supplementary file 1 [file metabolites-14-00272-s001.zip › 24.04.10-Supplementary tables.pdf]

Supplementary Tables

Table S1 Web of Science search equations and number of articles per search.

No.	Search commands using the field topic	No. ref.
1	Plant* AND （(Transcriptomics* OR “RNA Seq”） AND Metabolomics* ）	475
2	Plant* AND （(Transcriptomics* OR “RNA Seq”） AND Metabolomics* ） AND （(environment* OR ecotype*） AND (quality* OR character*))	46
3	Plant* AND （(Transcriptomics* OR “RNA Seq”） AND Metabolomics* ） AND （(environment* OR ecotype*） OR (quality* OR character*))	229
4	Plant* AND （(Transcriptomics* OR “RNA Seq”） OR Metabolomics* ） AND （(environment* OR ecotype*） AND (quality* OR character*))	576
5	Plant* AND （(Transcriptomics* OR “RNA Seq”） AND Metabolomics* ） AND （environment* OR ecotype* OR stress OR “environmental response”OR habitat* OR ecology*）	249
6	Plant* AND （(Transcriptomics* OR “RNA Seq”） AND Metabolomics* ） AND （quality* OR character* OR “active ingredient”OR “chemical marker”OR biosynthesis* OR “secondary metabolites”OR compound*)	269
7	Plant* AND （(Transcript* OR “RNA Seq”） AND Metabolite*） AND （(environment* OR ecotype*） AND (quality* OR character*))	234
8	Plant* AND （(Transcriptomics* OR “RNA Seq”OR “gene expression”OR “transcription factors”OR “Differentially expressed genes”） AND Metabolomics* ） AND （(environment* OR ecotype* OR stress） AND (quality* OR character*))	133
9	(Transcriptome* OR Transcriptomics* OR “RNA Seq”OR “gene expression”OR “transcription factors”OR “Differentially expressed genes”） AND （Metabolomics* OR metabolome* OR Metabolite*) AND （environment* OR ecotype* OR stress OR “environmental response”OR habitat* OR ecology*） AND （quality* OR character* OR “active ingredient”OR “chemical marker”OR biosynthesis* OR “secondary metabolites”OR compound*)	866
10	Plant* AND （(Transcriptomics* OR “RNA Seq”OR scRNA OR smFISH OR SSR OR SNP OR EST-SSR OR microRNA OR lncRNA OR “RNA editing” OR “Alternative splicing”） AND (Metabolomics* OR LC-MS OR NMR OR GC-MS OR CE-MS OR FT-IR ）)AND （ （environment* OR ecotype*） OR (quality* OR character*))	288
11	Plant* AND （Transcriptomics* OR “RNA Seq”OR scRNA OR smFISH OR SSR OR SNP OR EST-SSR OR microRNA OR lncRNA OR “RNA editing” OR “Alternative splicing”）	156
	In total	3521
	Total number of references without duplicates	1753

Table S2 The top clusters of co-occurrence in research on transcriptomics and metabolomics in plant quality and environmental response (TMPQE) during 1994-2020.

1994-2020				
ClusterID	Size	Silhouette	mean(Citee Year)	Label (LLR)
0	28	0.868	2008	arabidopsis leave
1	27	0.859	2010	maize pulvini
2	27	0.766	2009	specific function
3	25	0.838	2010	panicum virgatum
4	23	0.750	2009	secondary metabolite
2004-2015				
ClusterID	Size	Silhouette	mean(Citee Year)	Label (LLR)
0	31	0.692	2008	mitochondrial metabolism
1	28	0.589	2008	verticillium dahliae
5	22	0.685	2007	high temperature
2016-2020				
ClusterID	Size	Silhouette	mean(Citee Year)	Label (LLR)
0	17	0.839	2017	metabolome analyses
1	16	0.848	2017	candidate gene
3	14	0.82	2017	endogenous auxin level

Table S3 Leading journals in TMPQE research.

Ranked by TP				Ranked by TLC/t			
Rank	Journal	TP	TLCS/t	Rank	Journal	TP	TLCS/t
1	FRONTIERS IN PLANT SCIENCE	156	0	1	THE PLANT JOURNAL	109	45.48
2	PLANT PHYSIOLOGY	122	44.31	2	PLANT PHYSIOLOGY	122	44.31
3	BMC PLANT BIOLOGY	120	0	3	THE PLANT CELL	97	40.04
4	THE PLANT JOURNAL	110	45.48	4	JOURNAL OF EXPERIMENTAL BOTANY	77	18.25
5	PLANT CELL	97	40.04	5	PLANT CELL AND ENVIRONMENT	50	17.69
6	JOURNAL OF EXPERIMENTAL BOTANY	78	18.25	6	MOLECULAR PLANT	24	11.29
7	NEW PHYTOLOGIST	54	6.26	7	PLANT BIOTECHNOLOGY JOURNAL	35	8.3
8	PLANT PHYSIOLOGY AND BIOCHEMISTRY	52	5.43	8	PROCEEDINGS OF THE NATIONAL ACADEMY OF SCIENCES OF THE UNITED STATES OF AMERICA	8	7.22
9	PLANT CELL AND ENVIRONMENT	50	17.69	9	PLANT MOLECULAR BIOLOGY	39	6.85
10	BMC GENOMICS	42	0	10	NEW PHYTOLOGIST	53	6.26

Note: TP: total number of publication; TLCS/t: average local citations received per year.

Table S4 Journal Related Abbreviations.

Abbreviation	Explanation
PJ	THE PLANT JOURNAL
PP	PLANT PHYSIOLOGY
PC	THE PLANT CELL
JEB	JOURNAL OF EXPERIMENTAL BOTANY
PCE	PLANT CELL AND ENVIRONMENT
MP	MOLECULAR PLANT
PBJ	PLANT BIOTECHNOLOGY JOURNAL
PMB	PLANT MOLECULAR BIOLOGY
NP	NEW PHYTOLOGIST
PPB	PLANT PHYSIOLOGY AND BIOCHEMISTRY
PCP	PLANT AND CELL PHYSIOLOGY
PL	PLANTA
PH	PHYTOCHEMISTRY
EEB	ENVIRONMENTAL AND EXPERIMENTAL BOTANY
JPP	JOURNAL OF PLANT PHYSIOLOGY
PS	PLANT SCIENCE
FIPS	FRONTIERS IN PLANT SCIENCE
BPB	BMC PLANT BIOLOGY
BG	BMC GENOMICS
SR	SCIENTIFIC REPORTS

Table S5 Methodology related abbreviations.

Abbreviation	Explanation
TP	total number of publication
TLCS	total local citations received
TLCS/t	average local citations received per year
TGCS	total global citations received
TGCS/t	average global citations received per year
TLCR	total local cited references
h-index	an unbiased indicator to assess the performance of scientific outputs according to quantity and quality aspects
SCP	single country publication
MCP	multiple countries publication
MCP ratio	measure the international collaboration intensity of a country

Table S6 Theoretical basis: top 10 cited articles in research on transcriptomics and metabolomics in plant quality and environmental response (TMPQE).

No.	Title	Author	Year	Journal	Country	Institution	Citations	Classification	Ref.
#1	Analysis of relative gene expression data using real-time quantitative PCR and the 2(T)(-Delta Delta C) method	Livak KJ	2001	METHODS	USA	Washington State Univ	228	(a)	[44]
#2	Controlling the False Discovery Rate: A Practical and Powerful Approach to Multiple Testing	BENJAMINI Y	1995	JOURNAL OF THE ROYAL STATISTICAL SOCIETY SERIES B-STATISTICAL METHODOLOGY	Israel	Tei Aviv Univ	136	(a)	[45]
#3	MAPMAN: a user-driven tool to display genomics data sets onto diagrams of metabolic pathways and other biological processes	Thimm O	2004	PLANT JOURNAL	Germany	Max Planck Inst Mol Physiol	110	(b)	[46]
#4	Full-length transcriptome assembly from RNA-Seq data without a reference genome	Grabherr MG	2011	NATURE BIOTECHNOLOGY	Israel	Hebrew Univ Jerusalem	100	(b)	[47]
#5	Floral dip: a simplified method for Agrobacterium -mediated transformation of Arabidopsis thaliana	Clough SJ	1998	PLANT JOURNAL	USA	Department of Crop Sciences, University of Illinois at Urbana	93	(c)	[50]
#6	Blast2GO: a universal tool for annotation, visualization and analysis in functional genomics research	Conesa A	2005	BIOINFORMATICS	Spain	Inst Valenciano Invest Agr	81	(b)	[48]
#7	Gas chromatography mass spectrometry-based metabolite profiling in plants	Lisec J	2006	Nature Protocols	Germany	Max Planck Inst Mol Pflazenphysiol	79	(c)	[51]
#8	Mapping and quantifying mammalian transcriptomes by RNA-Seq	Mortazavi A	2008	NATURE METHODS	USA	Div Biol	77	(c)	[52]
#9	A Rapid and Sensitive Method for the Quantitation of Microgram Quantities of Protein Utilizing the Principle of Protein-Dye Binding	BRADFORD MM	1976	ANALYTICAL BIOCHEMISTRY	Georgia	Univ of Georgia	74	(c)	[53]

#10	Differential gene and transcript expression analysis of RNA-seq experiments with TopHat and Cufflinks	Trapnell C	2012	Nature Protocols	USA	Broad Inst MIT & Harvard	69	(b)	[49]

Note: Citations means the number of citations of the article in the TMPQE local dataset. (a) Theoretical algorithm; (b) Development of Bioinformatics tools and software; (c) Establishing experimental methods and protocol

Table S7 Detailed overview of the 29 most influential articles (with respect to TLC value > 14) from citation mapping

Node Number	Title	Year	Author	Journal	LCS	GCS	research streams group	Ref.
18	Integration of transcriptomics and metabolomics for understanding of global responses to nutritional stresses in Arabidopsis thaliana	2004	Hirai MY	PROCEEDINGS OF THE NATIONAL ACADEMY OF SCIENCES OF THE UNITED STATES OF AMERICA	43	469	(1)	[14]
19	Exploring the temperature-stress metabolome of Arabidopsis	2004	Kaplan F	PLANT PHYSIOLOGY	48	554	(2)	[69]
23	Functional genomics by integrated analysis of metabolome and transcriptome of Arabidopsis plants over-expressing an MYB transcription factor	2005	Tohge T	PLANT JOURNAL	33	569	(1)	[63]
24	Systems rebalancing of metabolism in response to sulfur deprivation, as revealed by metabolome analysis of arabidopsis plants	2005	Nikiforova VJ	PLANT PHYSIOLOGY	14	221	(1)	[57]
28	Elucidation of gene-to-gene and metabolite-to-gene networks in Arabidopsis by integration of metabolomics and transcriptomics	2005	Hirai MY	JOURNAL OF BIOLOGICAL CHEMISTRY	32	324	(1)	[58]
58	Omics-based identification of Arabidopsis Myb transcription factors regulating aliphatic glucosinolate biosynthesis	2007	Hirai MY	PROCEEDINGS OF THE NATIONAL ACADEMY OF SCIENCES OF THE UNITED STATES OF AMERICA (PNAS)	40	447	(1)	[66]
61	The R2R3-MYB transcription factor HAG1/MYB28 is a regulator of methionine-derived glucosinolate biosynthesis in Arabidopsis thaliana	2007	Gigolashvili T	PLANT JOURNAL	21	259	(1) & (3)	[67]
67	Transcriptional regulation of anthocyanin biosynthesis in ripening fruits of grapevine under seasonal water deficit	2007	Castellarin SD	PLANT CELL AND ENVIRONMENT	14	295	(1) & (4)	[80]
68	Water deficits accelerate ripening and induce changes in gene expression regulating flavonoid	2007	Castellarin SD	PLANTA	15	299	(1) & (2) & (4)	[68]

	biosynthesis in grape berries							
70	Quality control for plant metabolomics: reporting MSI-compliant studies	2008	Fiehn O	PLANT JOURNAL	14	326	(5)	[83]
92	Metabolic responses to salt stress of barley (Hordeum vulgare L.) cultivars, Sahara and Clipper, which differ in salinity tolerance	2009	Widodo	JOURNAL OF EXPERIMENTAL BOTANY	17	204	(2)	[70]
107	Ectopic Expression of VvMybPA2 Promotes Proanthocyanidin Biosynthesis in Grapevine and Suggests Additional Targets in the Pathway	2009	Terrier N	PLANT PHYSIOLOGY	16	207	(1)	[64]
113	Characterization of the ABA-regulated global responses to dehydration in Arabidopsis by metabolomics	2009	Urano K	PLANT JOURNAL	30	287	(2)	[75]
137	A guide to using MapMan to visualize and compare Omics data in plants: a case study in the crop species, Maize	2009	Usadel B	PLANT CELL AND ENVIRONMENT	21	300	(5)	[81]
165	The Arabidopsis bZIP transcription factor HY5 regulates expression of the PFG1/MYB12 gene in response to light and ultraviolet-B radiation	2010	Stracke R	PLANT CELL AND ENVIRONMENT	15	182	(2)	[71]
166	Developmental Stage Specificity and the Role of Mitochondrial Metabolism in the Response of Arabidopsis Leaves to Prolonged Mild Osmotic Stress	2010	Skirycz A	PLANT PHYSIOLOGY	14	155	(2)	[76]
183	Characterization of Transcriptional Complexity during Berry Development in Vitis vinifera Using RNA-Seq	2010	Zenoni S	PLANT PHYSIOLOGY	14	248	(1) & (4)	[61]
228	Molecular and Physiological Analysis of Drought Stress in Arabidopsis Reveals Early Responses Leading to Acclimation in Plant Growth	2010	Harb A	PLANT PHYSIOLOGY	14	329	(2)	[73]
255	Negative Regulation of Anthocyanin Biosynthesis in Arabidopsis by a miR156-Targeted SPL Transcription Factor	2011	Gou JY	PLANT CELL	16	352	(1)	[65]
260	The Jasmonate-ZIM-Domain Proteins Interact with the WD-Repeat/bHLH/MYB Complexes to Regulate Jasmonate-Mediated Anthocyanin Accumulation	2011	Qi TC	PLANT CELL	15	354	(1)	[88]

	and Trichome Initiation in Arabidopsis thaliana							
330	Drought Responses of Leaf Tissues from Wheat Cultivars of Differing Drought Tolerance at the Metabolite Level	2012	Bowne JB	MOLECULAR PLANT	16	165	(2)	[74]
383	The Grapevine Expression Atlas Reveals a Deep Transcriptome Shift Driving the Entire Plant into a Maturation Program	2012	Fasoli M	PLANT CELL	17	178	(1) & (4)	[59]
401	Genome-Wide Analysis Uncovers Regulation of Long Intergenic Noncoding RNAs in Arabidopsis	2012	Liu J	PLANT CELL	14	305	(5)	[84]
444	Transcriptome Responses to Combinations of Stresses in Arabidopsis	2013	Rasmussen S	PLANT PHYSIOLOGY	15	213	(5)	[85]
459	Comprehensive Dissection of Spatiotemporal Metabolic Shifts in Primary, Secondary, and Lipid Metabolism during Developmental Senescence in Arabidopsis	2013	Watanabe M	PLANT PHYSIOLOGY	14	136	(1) & (4)	[60]
467	Arabidopsis Basic Helix-Loop-Helix Transcription Factors MYC2, MYC3, and MYC4 Regulate Glucosinolate Biosynthesis, Insect Performance, and Feeding Behavior	2013	Schweizer F	PLANT CELL	15	189	(1) & (3)	[77]
522	Enhancement of oxidative and drought tolerance in Arabidopsis by overaccumulation of antioxidant flavonoids	2014	Nakabayashi R	PLANT JOURNAL	28	273	(1) & (2)	[72]
552	Mercator: a fast and simple web server for genome scale functional annotation of plant sequence data	2014	Lohse M	PLANT CELL AND ENVIRONMENT	18	218	(5)	[82]
713	Full-length transcriptome sequences and splice variants obtained by a combination of sequencing platforms applied to different root tissues of Salvia miltiorrhiza and tanshinone biosynthesis	2015	Xu ZC	PLANT JOURNAL	18	124	(1)	[62]

Note: research streams group: (1) biosynthetic pathway; (2) abiotic stress; (3) biotic stress; (4) development and ripening; (5) methodology and tools.
